# A tribute to John Q. Trojanowski (1946–2022)

**DOI:** 10.1172/JCI161019

**Published:** 2022-05-16

**Authors:** David J. Irwin, Edward B. Lee

**Affiliations:** 1 Department of Neurology and; 2 Department of Pathology and Laboratory Medicine, Perelman School of Medicine, University of Pennsylvania, Philadelphia, Pennsylvania, USA.

We unfortunately suffered the loss of an iconic figure in the field of neurodegenerative diseases when Dr. John Q. Trojanowski, MD, PhD, passed away on February 8, 2022, at the age of 75 (Figure 1). John’s scientific contributions to the study of Alzheimer’s disease (AD) and related neurodegenerative disorders (ADRD; i.e., dementia with Lewy bodies [DLB], Parkinson’s disease [PD], frontotemporal lobar degeneration [FTLD], and amyotrophic lateral sclerosis [ALS]) span more than four decades. His seminal discoveries range from providing fundamental mechanistic insights into the main protein constituents of the characteristic inclusions of these disorders (1–3), leading major efforts to develop biofluid and imaging biomarkers for AD (4, 5), and championing the concept of mixed pathology in aging (6) through building an extensive brain bank of more than 2000 well-annotated and -curated patient samples (7) that are shared throughout the world to advance research in ADRD. John’s scientific accomplishments and influence within any of these main areas alone would be considered a highly successful career as a physician-scientist; in totality, they represent a truly remarkable and unparalleled body of work. Together with his wife and colleague, Dr. Virginia Lee, he developed a powerhouse of multidisciplinary science at the Penn Center for Neurodegenerative Disease Research (CNDR). Those who are fortunate to have been trained at CNDR by John share a common bond of experiencing enormous opportunity and unwavering support from a scientist of immense stature who simultaneously projected a warm and welcoming demeanor.

John was born in 1946 in Bridgeport, Connecticut, and moved several times within the United States and overseas during his childhood due to his father’s career as an Air Force officer. John began his education with a full scholarship to King’s College in Pennsylvania, graduating in 1970 with a major in German studies. During this time, he also studied abroad, in both the Netherlands and Austria; it was not uncommon for John to demonstrate his multilingualism during lab meetings while recounting a tale of his time in Vienna, which brought a lightening and playful tone to the discussions. John obtained his medical and doctoral degrees in 1976 at Tufts University in Boston, where his initial work focused on the neuroanatomy of the pulvinar nucleus of thalamus in nonhuman primates (8–12). John meticulously curated a collection of paper and electronic copies of manuscripts in his office; in casual conversation, he would frequently fetch the perfect article from this collection to help a trainee formulate the next step in their planned experiments or direct a colleague to an important resource for future studies. Indeed, he would often fondly recall the early neuroanatomic studies of his PhD days and still had the paper copies of these ready for discussions on tau propagation in the human brain.

After his medical school training, John completed internal medicine and neuropathology training at Massachusetts General Hospital (MGH). One striking characteristic of John was his curiosity and eagerness to engage with others in different scientific disciplines. This is perhaps most exemplified by his pursuit of a full year of internal medicine training prior to subspecialization in neuropathology. John would recall how much he enjoyed spending time seeing patients during his training at MGH, and the connection he had to clinical medicine remained throughout his career as a neuropathologist. In a time when clinical syndromic definitions of AD and related disorders lacked a clear biological cause, John brought pathology to life and engaged with neurology and psychiatry colleagues to make neuropathology tissue and data accessible to both neuropathologists and those outside the field. Indeed, he trained several postdocs from a clinical neurology background in experimental neuropathology, recognizing the importance of advancing knowledge of the neuropathology of ADRD. Perhaps one of his most underappreciated innovations was the pioneering of the Penn Integrated NeuroDegenerative Disease Database (7), where his expert ratings of pathological severity across brain regions were not only used for diagnosis but systematically stored as data and linked to antemortem clinical, imaging, genetic, and biofluid biomarker data. By doing so, John created a tremendous resource of neuropathology data and made it available for neurodegenerative disease researchers from varying backgrounds at Penn and beyond. Thus, he transformed the narrative of the clinical autopsy report into a quantitative and powerful research tool.

It was at MGH where he met Virginia. Together they were recruited to the University of Pennsylvania in 1981. In less than a decade, John was promoted through the ranks to full professor in 1990 and led his first program project grant on AD and PD. John spent the remainder of his career at Penn, where he led a highly successful NIH Alzheimer’s Disease Core Center for over 20 years, as well as program projects on PD and LBD, and directed the Penn Institute on Aging. In this capacity, John created a very productive infrastructure of neurodegenerative disease research that included faculty recruitments across the neuropathology, neurology, psychiatry, and medicine departments at Penn. Importantly, he grew these programs with a personal style and created a family-like atmosphere. John could be reached almost instantly by email but often preferred to pick up his phone to maintain contact with various colleagues across ongoing programs and projects. John had numerous awards and professional and honorary memberships, including being a longstanding member of the American Society for Clinical Investigation and the National Academy of Medicine. Despite his many leadership roles, John maintained a steady calmness and impressive level of organization, sometimes simply by carrying a folded piece of paper to a meeting to jot down notes for follow-up later.

John and Virginia blended a highly potent approach combining neuropathology and biochemistry, leading to their first major discovery in 1991, when they isolated paired helical filaments from AD brains and identified tau as the protein constituent of neurofibrillary tangle pathology (1). Today, tau pathology is detected in cerebrospinal fluid and positron imaging in living patients for diagnosis of AD and is a target for several ongoing clinical trials in AD and other tauopathies. Indeed, together with Dr. Leslie Shaw, John also made tremendous contributions to advances in identifying and validating biofluid biomarkers to detect AD pathology in living patients in the first large-scale public-private collaborative multicenter efforts to study AD (ref. 4; i.e., Alzheimer’s Disease Neuroimaging Initiative [ADNI]) and PD (Parkinson’s Progression Markers Initiative [PPMI]; ref. 13). In 1997 mutations in the *SNCA* gene, encoding the α-synuclein protein, were identified as a cause of familial PD and DLB (14).

In collaboration with Dr. Maria Spillantini and Dr. Michel Goedert, in 1997 John harnessed the CNDR brain bank to probe sporadic human brain PD and DLB tissues for α-synuclein immunoreactivity and found Lewy bodies in both disorders to contain α-synuclein (2), which has led to our current understanding of both disorders as α-synucleinopathies. While roughly 50% of frontotemporal lobar degeneration was revealed to be caused by non-AD tau pathology (FTLD-Tau) from John and Virginia’s earlier work, there remained a large number of patients with clinical frontotemporal dementia syndromes with postmortem findings of ubiquitinated protein inclusions of unclear etiology or “dementia lacking distinctive histopathology.” In seminal work requiring complex biochemistry to isolate and characterize these ubiquitinated inclusions, John and Virginia discovered the protein TDP-43 as the characteristic inclusion in these non-tauopathy forms of FTLD (now known as FTLD-TDP) in 2006 (3). Remarkably, TDP-43 was also found in over 90% of patients with ALS, linking these two seemingly distinct clinical dementia and neuromuscular syndromes into what is now recognized as a clinicopathological spectrum with shared genetic risk (15).

More recently, John utilized the brain bank at Penn to study TDP-43 in the context of other neurodegenerative disorders, including findings of clinically relevant TDP-43 pathology in AD (6), corticobasal degeneration (16), Alexander’s disease (17), and LBD (18). Finally, John and Virginia have successfully modeled tau (19), α-synuclein (20), and TDP-43 (21) proteinopathies in innovative animal and cell models, which demonstrated that abnormal misfolding and propagation of these proteins alone can recapitulate human disease. The impact of these discoveries and subsequent work, representing more than 1400 publications, cannot be understated, and their influence will remain for years to come.

Another of John’s most memorable and endearing attributes was the way he welcomed all to learn from him and his ability to listen and instill confidence in his trainees. The multi-head microscope at CNDR remains a fixture that is emblematic of John’s wonderful character. It was not uncommon during review of a diagnostic case for John to call over others to review slides with him, regardless of their stage of training. An undergraduate summer student had an equal opportunity to share the microscope as a seasoned senior postdoc, and all in his lab had the privilege of sharing time with John and asking questions as he pointed out nuances of neuropathology — an experience available to few in the world. Despite being in high demand at scientific meetings, we both have experienced the remarkably meaningful gesture of John seeking us out to introduce to other leaders in the field.

John was energetic, with an infectious spirit of enthusiasm, and he was highly motivated to find a cure for Alzheimer’s disease and related neurodegenerative disorders. John’s scientific legacy is a foundation that much of current ADRD research stands upon. While we have not realized this goal during his lifetime, the charge is upon all of us in the field to build on his seminal contributions and the work of others toward this ultimate goal for humanity. We celebrate a dear friend and mentor to many across the globe who is dearly missed but not forgotten.

## Figures and Tables

**Figure 1:**
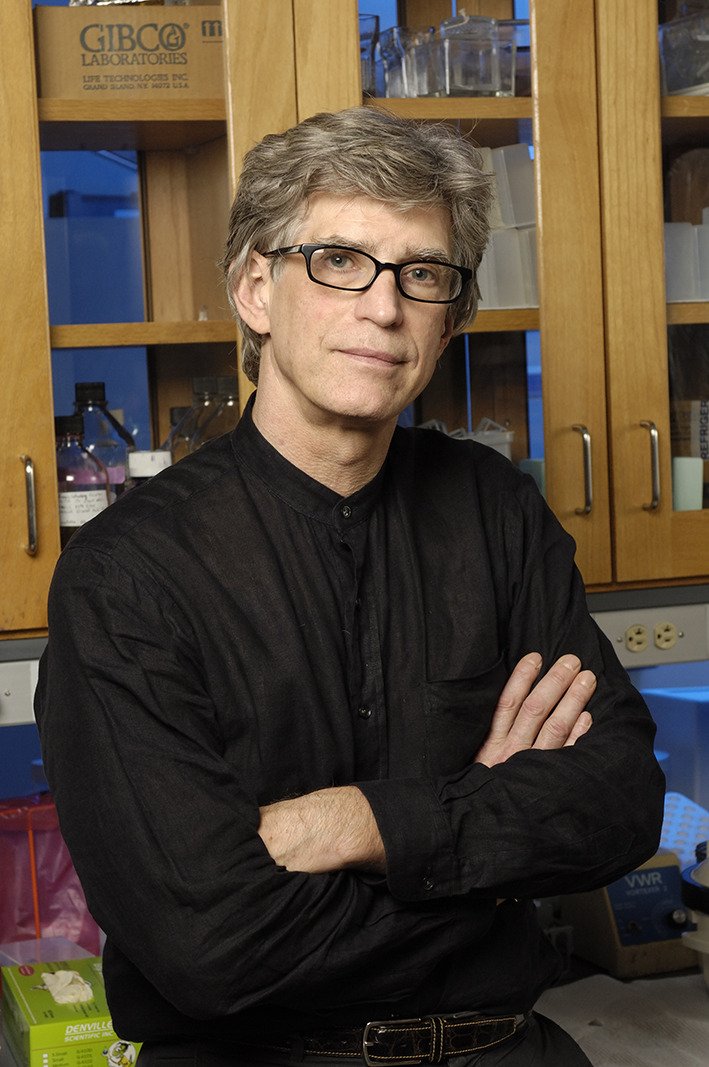
John Q. Trojanowski, MD, PhD. Photo courtesy of the Office of University Communications, University of Pennsylvania.

## Version 1 05/16/2022

Electronic publication

## References

[1] Lee VM (1991). A68: a major subunit of paired helical filaments and derivatized forms of normal Tau. Science.

[2] Spillantini MG (1997). Alpha-synuclein in Lewy bodies. Nature.

[3] Neumann M (2006). Ubiquitinated TDP-43 in frontotemporal lobar degeneration and amyotrophic lateral sclerosis. Science.

[4] Shaw LM (2009). Cerebrospinal fluid biomarker signature in Alzheimer’s disease neuroimaging initiative subjects. Ann Neurol.

[5] Skovronsky DM (2000). In vivo detection of amyloid plaques in a mouse model of Alzheimer’s disease. Proc Natl Acad Sci U S A.

[6] Robinson JL (2018). Neurodegenerative disease concomitant proteinopathies are prevalent, age-related and APOE4-associated. Brain.

[7] Toledo JB (2013). A platform for discovery: The University of Pennsylvania Integrated Neurodegenerative Disease Biobank. Alzheimers Dement.

[8] Trojanowski JO, Jacobson S (1975). Peroxidase labeled subcortical afferents to pulvinar in rhesus monkey. Brain Res.

[9] Trojanowski JQ, Jacobson S (1974). Medial pulvinar afferents to frontal eye fields in rhesus monkey demonstrated by horseradish peroxidase. Brain Res.

[10] Trojanowski JQ, Jacobson S (1975). A combined horseradish peroxidase-autoradiographic investigation of reciprocal connections between superior temporal gyrus and pulvinar in squirrel monkey. Brain Res.

[11] Trojanowski JQ, Jacobson S (1976). Areal and laminar distribution of some pulvinar cortical efferents in rhesus monkey. J Comp Neurol.

[12] Trojanowski JQ, Jacobson S (1977). The morphology and laminar distribution of cortico-pulvinar neurons in the rhesus monkey. Exp Brain Res.

[13] Kang JH (2013). Association of cerebrospinal fluid Aβ1-42, t-tau, p-tau181 and α-synuclein levels with clinical features of early drug naïve Parkinson’s disease patients. JAMA Neurol.

[14] Polymeropoulos MH (1997). Mutation in the alpha-synuclein gene identified in families with Parkinson’s disease. Science.

[15] Lee EB (2012). Gains or losses: molecular mechanisms of TDP43-mediated neurodegeneration. Nat Rev Neurosci.

[16] Uryu K (2008). Concomitant TAR-DNA-binding protein 43 pathology is present in Alzheimer disease and corticobasal degeneration but not in other tauopathies. J Neuropathol Exp Neurol.

[17] Walker AK (2014). Astrocytic TDP-43 pathology in Alexander disease. J Neurosci.

[18] Uemura MT (2022). Distinct characteristics of limbic-predominant age-related TDP-43 encephalopathy in Lewy body disease. Acta Neuropathol.

[19] Iba M (2013). Synthetic tau fibrils mediate transmission of neurofibrillary tangles in a transgenic mouse model of Alzheimer’s-like tauopathy. J Neurosci.

[20] Luk KC (2012). Intracerebral inoculation of pathological α-synuclein initiates a rapidly progressive neurodegenerative α-synucleinopathy in mice. J Exp Med.

[21] Porta S (2018). Patient-derived frontotemporal lobar degeneration brain extracts induce formation and spreading of TDP-43 pathology in vivo. Nat Commun.

